# Defining type 2 diabetes polygenic risk scores through colocalization and network-based clustering of metabolic trait genetic associations

**DOI:** 10.1186/s13073-023-01255-7

**Published:** 2024-01-10

**Authors:** Samuel Ghatan, Jeroen van Rooij, Mandy van Hoek, Cindy G. Boer, Janine F. Felix, Maryam Kavousi, Vincent W. Jaddoe, Eric J. G. Sijbrands, Carolina Medina-Gomez, Fernando Rivadeneira, Ling Oei

**Affiliations:** 1 Department of Internal Medicine, Erasmus MC University Medical Center Rotterdam, Rotterdam, The Netherlands; 2 The Generation R Study Group, Erasmus MC, Erasmus University Medical Center Rotterdam, Rotterdam, The Netherlands; 3 Department of Pediatrics, Erasmus MC, Erasmus University Medical Center Rotterdam, Rotterdam, The Netherlands; 4 Department of Epidemiology, Erasmus MC University Medical Center Rotterdam, Rotterdam, The Netherlands

**Keywords:** Polygenic risk score, Type 2 diabetes, Colocalization, Clustering, Personalized medicine

## Abstract

**Background:**

Type 2 diabetes (T2D) is a heterogeneous and polygenic disease. Previous studies have leveraged the highly polygenic and pleiotropic nature of T2D variants to partition the heterogeneity of T2D, in order to stratify patient risk and gain mechanistic insight. We expanded on these approaches by performing colocalization across GWAS traits while assessing the causality and directionality of genetic associations.

**Methods:**

We applied colocalization between T2D and 20 related metabolic traits, across 243 loci, to obtain inferences of shared casual variants. Network-based unsupervised hierarchical clustering was performed on variant-trait associations. Partitioned polygenic risk scores (PRSs) were generated for each cluster using T2D summary statistics and validated in 21,742 individuals with T2D from 3 cohorts. Inferences of directionality and causality were obtained by applying Mendelian randomization Steiger’s *Z*-test and further validated in a pediatric cohort without diabetes (aged 9–12 years old, *n* = 3866).

**Results:**

We identified 146 T2D loci that colocalized with at least one metabolic trait locus. T2D variants within these loci were grouped into 5 clusters. The clusters corresponded to the following pathways: obesity, lipodystrophic insulin resistance, liver and lipid metabolism, hepatic glucose metabolism, and beta-cell dysfunction. We observed heterogeneity in associations between PRSs and metabolic measures across clusters. For instance, the lipodystrophic insulin resistance (Beta − 0.08 SD, 95% CI [− 0.10–0.07], *p* = 6.50 × 10^−32^) and beta-cell dysfunction (Beta − 0.10 SD, 95% CI [− 0.12, − 0.08], *p* = 1.46 × 10^−47^) PRSs were associated to lower BMI. Mendelian randomization Steiger analysis indicated that increased T2D risk in these pathways was causally associated to lower BMI. However, the obesity PRS was conversely associated with increased BMI (Beta 0.08 SD, 95% CI 0.06–0.10, *p* = 8.0 × 10^−33^). Analyses within a pediatric cohort supported this finding. Additionally, the lipodystrophic insulin resistance PRS was associated with a higher odds of chronic kidney disease (OR 1.29, 95% CI 1.02–1.62, *p* = 0.03).

**Conclusions:**

We successfully partitioned T2D genetic variants into phenotypic pathways using a colocalization first approach. Partitioned PRSs were associated to unique metabolic and clinical outcomes indicating successful partitioning of disease heterogeneity. Our work expands on previous approaches by providing stronger inferences of shared causal variants, causality, and directionality of GWAS variant-trait associations.

**Supplementary Information:**

The online version contains supplementary material available at 10.1186/s13073-023-01255-7.

## Background

Type 2 diabetes (T2D) is a common heterogeneous disease, affecting over 400 million individuals worldwide. The underlying complexity of T2D goes beyond the simple measure of blood glucose used for diagnosis. The regulation of glucose metabolism has many diverse components, contributing to this heterogeneity [1] observed in the clinical presentation of the disease. It is of interest to gain a deeper understanding of the heterogeneity of T2D to potentially improve patient care through the development of targeted treatments and improving diagnosis [2, 3]. Genetic variants identified via genome-wide association studies (GWAS), in the form of polygenic risk scores (PRSs), can serve to disentangle this heterogeneity and can provide valuable tools for risk prediction. Currently, GWAS have identified hundreds of genetic variants associated with T2D risk. These variants map to different genes and have been annotated to multiple distinct pathways linked to T2D [4]. A promising avenue to gain biological insights from GWAS signals is to leverage the highly pleiotropic nature of genetic variants associated with T2D and a plethora of other traits [5]. Pleiotropy is the property of genetic variants affecting multiple traits and is now recognized to be widespread across the genome [5, 6]. Variants that display similar associations with intermediary phenotypes can be annotated to the same pathway. This approach was first used to establish the polygenic effect of common variants acting through the lipodystrophic insulin resistance pathway [7, 8]. Recent studies using this approach identified five to six clusters representing biological pathways of T2D that associated with specific clinical measures and outcomes in individuals with T2D [4, 9–11]. However, such annotation of variants to pathways and T2D clusters is not free of limitations. Investigating whether two or more GWAS share the same causal variant is a complex undertaking [12]. The existence of linkage disequilibrium (LD) between potential causal and non-causal marker variants introduces a challenge, as even if a variant exhibits a significant association with multiple GWAS, distinct causal variants might underlie these associations. This problem is further exacerbated by the high polygenicity of complex traits, as unraveled by the increasingly larger sample sizes of GWAS [13]. Moreover, in order to infer evidence regarding potential shared causal variants among two or more GWAS, a formal test of a null hypothesis must be undertaken. Even if it can be established that two traits are influenced by a common causal variant, determining the directionality of the association introduces an additional hurdle. This is due to the ambiguity surrounding whether one trait acts as a mediator for the other, or if the causal variant independently affects them both. Past endeavors fell short in addressing these intricacies while evaluating the pleiotropic impacts of T2D variant. In this study, we sought to build on previous approaches in establishing whether T2D genetic variants can be grouped according to their shared pleiotropic associations. To achieve this, we employed colocalization analysis as a foundational step, enabling us to derive more robust inferences regarding pleiotropic associations. Additionally, network analysis was carried out to identify clusters of genetic variants that exhibit shared pleiotropic associations. To evaluate whether these clusters represent biological pathways underlying T2D risk, we constructed partitioned [9, 11, 14] polygenic risk scores (PRSs) using T2D genetic risk variants for each cluster. PRSs were tested against relevant measures of metabolism and clinical outcomes in 21,742 individuals with T2D across three cohorts (UK biobank, the Rotterdam Study and the DiaGene Study). To assess the directionality and causality of the generated clusters, we performed directionality tests and Mendelian randomization. Lastly, we validated these findings in a pediatric cohort aged 9–12 years old (*n* = 3866) in relation to glycemic traits in children without diabetes.

## Methods

### Study populations

#### The UK Biobank

The study design and methods of the UK Biobank (UKB) have been reported previously [15]. Shortly, the UKB is a prospective cohort study within the United Kingdom (UK), recruiting approximately 500,000 individuals aged between 40 and 69, across multiple sites throughout the UK. For the current study, only T2D cases of European descent were included (*n* = 17,741). UKB data was accessed using application number 67864. Study descriptions can be found in Additional file 1: Table S1.

#### The DiaGene Study

DiaGene is a case–control study of T2D conducted in Eindhoven, the Netherlands, between 2006 and 2011 [16]. The study consists of 1886 T2D individuals and 854 non-T2D controls. T2D cases were recruited from both primary and secondary care. For the current study, only T2D cases of European descent were included (*n* = 1760). Study descriptives can be found in Additional file 1: Table S1 and measurement details in the Additional file 2.

#### The Rotterdam Study

The Rotterdam Study is a population-based cohort study that started in 1990, comprising about 14,926 participants aged 55 years or over, with the aim of studying chronic diseases in the general population [17]. The Rotterdam Study comprises one original cohort (RS-I, initiated in 1990, 7983 participants aged 55 years and over) and two other cohorts (RS-II, starting from 2000, 3011 participants aged 55 years and over; and RS-III, starting from 2006, 3,932 aged 45 years and over). We used all available diabetes data collected between 1997 and 2015 for this study. For the current study, only T2D cases of European descent were included (*n* = 2214). Study descriptives can be found in Additional file 1: Table S1 and measurement details in the Additional file 2.

#### The Generation R Study

The Generation R Study is a multi-ethnic prospective cohort study in which 9778 pregnant women living in Rotterdam and with delivery date from April 2002 until January 2006 were enrolled. Study design and data collection details can be found elsewhere [18]. Genotype and imputation of this cohort are described elsewhere [19]. In total, 3866 children aged between 9 and 12 years old of European descent and with genotype, BMI and fat percentage data were included in the study. Study descriptives can be found in Additional file 1: Table S1 and measurement details in the Additional file 2.

#### Diabetes definition

Within the UKB T2D was defined as individuals who self-reported having T2D at study recruitment. Additionally, individuals who received an International Statistical Classification of Diseases and Related Health Problems (ICD) version 10 code (E11-E14, defined as “non-insulin-dependent diabetes mellitus”) before the recruitment date were included. ICD-10 codes were obtained via electronic health records. Individuals with a type 1 diabetes ICD-10 code (E10) were excluded from the analysis. Within the DiaGene Study, information on T2D diagnosis was obtained through patient medical records. In accordance with the American Diabetes Association and World Health Organization guidelines, diabetes was defined as fasting plasma glucose ≥ 7.0 mmol/L and/or a non-fasting plasma glucose level ≥ 11.1 mmol/L measured at least at two different time points, treatment with oral glucose-lowering medication or insulin, and/or a diagnosis of T2D as registered by a medical specialist. Individuals diagnosed with type 1 diabetes (as derived from medical records and patient-questionnaires) or other types of diabetes mellitus were excluded from the study. Within the RS T2D status was ascertained through active follow-up using general practitioners’ records, glucose in hospital discharge letters, and glucose measurements from the Rotterdam Study visits. T2D was defined as fasting blood glucose > 7.0 mmol/L, or the use of blood-glucose-lowering medication. Information regarding the use of glucose-lowering medication was derived from both structured home interviews and linkage to pharmacy records.

#### Genotyping

Genotyping within the UKB was conducted using a custom UK Biobank Axiom genotype panel. Genotypes were imputed using a combination of the 1000G phase 3 and UK10K reference panels, as previously reported [15]. Within the DiaGene Study, participants were genotyped using the GSA array and imputed haplotype reference consortium (HRC) r1.1 reference panel [20]. Within the Rotterdam Study, RS1 and RS2 were genotyped using the Illuminia HumanHap 550 k genotyping array and RS3 was genotyped using a combination of Illuminia HumanHap 550 k and 610 k. All three RS cohorts were imputed using the HRC r1.1 reference panel. Within the Generation R Study, genotyping was performed using Illumina Human 610 k, 660W, and GSA V2 arrays. Genotype data were imputed to 1000G phase 3 v5 [21].

#### GWAS data

T2D GWAS summary statistics and conditionally independent lead variants were obtained from Mahajan et al. (without the adjustment for BMI and excluding UKB samples) [4]. T2D summary statistics can be obtained from DIAGRAM consortium website (https://diagram-consortium.org/downloads.html). Metabolic trait GWAS for colocalization were selected based on the following criteria: whether the trait (i) is an established clinical risk factor for T2D [22], (ii) is known to be involved in T2D pathophysiology [23], and (iii) has a significant genetic correlation with T2D [4, 24]. GWAS summary statistics with larger sample sizes and/or newer imputation reference panels were prioritized. Other considerations made when selecting traits were favoring those with more well-established pathophysiological mechanisms to T2D over those with ambiguous or bi-directional relationships to ensure robust associations [25]. Twenty metabolic traits were selected. These included alanine aminotransferase (ALT), gamma-glutamyl transferase (GGT) [26], high-density lipoprotein (HDL) cholesterol, triglycerides, low-density lipoprotein (LDL) cholesterol [27], visceral adipose tissue [28], arm fat ratio, leg fat ratio, trunk fat ratio [29], waist-to-hip ratio, body mass index [30], proinsulin levels [31], HOMA-IR [32], insulin sensitivity index [33], leptin (adjusted for BMI) [34], adiponectin [35], HbA_1C_, fasting glucose, fasting insulin, and 2-h glucose tolerance test values [36]. In sensitivity analysis oral glucose tolerance test (OGTT) [37] was also tested for colocalization with T2D (see Additional file 3). The HOMA-IR indicates the extent of insulin resistance, i.e., when target tissues do not respond sensitively to insulin and cannot easily take up glucose from the blood; a HOMA-IR of greater than 1.0 means that an individual is more insulin resistant, which is associated with diabetes. All clinical outcomes in adult cohorts were standardized to have a mean zero and standard deviation of one. A detailed table containing the characteristics of the metabolic trait GWAS used in this study can be found in Additional file 1: Table S2.

### Statistical methods

#### Colocalization

Colocalization is a statistical approach for assessing whether pairs of traits share a putative causal variant in the same region of the genome [12]. Briefly, the method adopts a Bayesian approach to enumerate over all variant-level hypotheses and assess the support for each using Bayes factors calculated from SNP effect estimates and standard errors. These variant-level hypotheses correspond to the following global hypotheses: H0: no association to either trait in the region; H1: association to only trait 1; H2: association to only trait 2; H3: associations to both traits, yet, with different causal variants; and H4: associations to both traits and causal variants are the shared. Summing the log Bayes factor for each global hypothesis and combining with prior probabilities allows for the calculation of posterior probabilities. In this description, each region is assumed to contain only one causal variant. However, this has been expanded to allow multiple causal variants by first determining putatively causal variants within a region using Bayesian stepwise regression [38]. Mahajan reported 243 loci containing SNPs at genome-wide significance (5 × 10^−8^), containing 403 conditionally independent lead variants (identified via GCTA approximate conditional analysis), associated with T2D [4]. A 1Mbp (500 kb on either side) region around the lead SNP in each locus was then defined. For a detailed explanation of the colocalization workflow see Additional file 2. Summary statistics were extracted from these regions across all metabolic trait GWAS. If a particular defined region contained just one T2D conditionally independent SNP, the probability of colocalization was tested using Hyprcoloc across all traits simultaneously [39]. A genomic region across traits was deemed to have significant evidence of colocalization when the overall posterior probability (P_R_P_A_) > 0.60 [39] and a variant-trait association *p*-value of < 1 × 10^−5^. Hyprcoloc was not able to investigate multiple causal variants in a locus; therefore, T2D regions containing multiple causal variants were investigated further using the Sum of Single Effects (SuSiE) coloc framework [38, 40]. Regions with P_R_ > 0.8 but without significant evidence of colocalization (P_R_P_A_) < 0.60 were investigated using SuSiE. Utilizing Hyprcoloc as a first stage of analysis and for prioritizing regions for SuSiE reduced the number of pairwise test by 3473, compared to if SuSiE alone was used. Variants were deemed to have colocalized if the posterior probability of hypothesis 4 (H4) > 0.6, where H4 relates to the hypothesis that the same variant is associated with both trait 1 and trait 2. In some situations, SuSiE was unable to identify any credible sets for a particular genomic region in which case COLOC [12] was applied under the single causal variant assumption. In the event of significant colocalization between T2D and a metabolic trait, the T2D lead casual variant within the colocalized credible set defined by SuSiE was extracted from the metabolic trait GWAS summary statistics. SNPs were then aligned to the effect-increasing allele. Palindromic SNPs with ambiguous allele frequencies (0.40 < minor allele frequency < 0.60) were removed and replaced with proxies with high LD (r2 > 0.8).

#### Network clustering

Network-based hierarchical clustering was performed to identify homogenous groups of variants in the observed pleiotropic associations. This approach has two advantages over soft clustering approaches. It allows the use of sparse matrices and avoids the problem of defining an arbitrary cluster assignment cut-off value [41]. A sparse matrix of colocalized SNP-trait associations (*Z*-scores) was constructed using variant-trait associations. Only variant-trait associations with a *p*-value less than 5 × 10^−8^ (for GWAS sample size greater than 60,000) and 1 × 10^−5^ (for GWAS sample size less than 60,000) were kept. A value of 0 was assigned to the remaining variant-trait associations that showed no statistically significant evidence of colocalization. This correlation matrix was then used to create a network map of the relationships between variants. In order to reduce noise when clustering, network edges with low correlations were removed as described hereafter. An appropriate correlation cut-off was determined by finding the highest cut-off point at which all vertexes still had at least one edge between them; indicating the network was still connected. A reasonable assumption considering that all variants within the network colocalized with at least one other trait and the traits are highly correlated. The correlation cut-off was determined to be 0.47 at which point all vertexes still had at least one edge. Network were constructed and clustered using the igraph package in R [42].

#### Gene Ontology (GO) annotations

We concentrated on GO biological process terms from the GO database [43]. Variants were mapped to their closest protein-coding genes. Next, these gene sets were tested for enrichment using the g:GOst enrichment test from the g:Profiler R package [44]. All protein-coding genes were used as a background set for the enrichment tests.

#### Polygenic risk scores

The overall T2D PRS consisted of all T2D variants that colocalized with at least one trait and clustered into one of 10 clusters (143 SNPs). Five clusters contained three genetic variants or less and were excluded from the main results (see Additional file 3). PRSs were generated for the remaining five clusters (135 SNPs). These five partitioned PRSs corresponded to the following clusters: beta-cell dysfunction (28 SNPs), hepatic glucose metabolism (6 SNPs), lipodystrophic insulin resistance (52 SNPs), liver and lipid metabolism (9 SNPs), and obesity (40 SNPs). The SNP effect sizes used to construct PRSs for the Rotterdam Study, DiaGene, and Generation R Study cohorts, were obtained from the T2D BMI-unadjusted summary statistics published by Mahajan et al. [4]. In the UKB cohort, SNP effect sizes were obtained from meta-analysis results not containing the UK biobank cohort and unadjusted by BMI. Both summary statistics are available for download on the Diagram website (https://diagram-consortium.org/). PRSs for each cluster of variants were calculated by multiplying the genotype dosage of each risk allele for each variant by its respective effect in the meta-analysis, summing across all variants for each participant. For the Rotterdam Study, DiaGene and Generation R cohorts PRSs were calculated from variant dosages. For the UKB cohort, PRSice2 was used to calculate the PRS [45]. All scores were standardized (scaled and centered) using the mean and standard deviation of all scores. Palindromic SNPs were removed and replaced with proxies. PRSs were tested using a linear regression (controlling for age, sex, BMI, and cohort, or age, sex, and cohort in the case of BMI as the outcome) for continuous metabolic outcomes and logistic regression for binary clinical outcomes. To test the potential effect of medication on metabolic levels, sensitivity analysis was conducted in which lipid-lowering and anti-diabetic medication were included as covariates in the regression models (Additional file 2). The significance level was corrected for the number of PRSs tested using the Bonferroni multiple testing correction. In the three adult cohorts, this amounted to 0.05/6 = 0.008 and for the pediatric cohort 0.05/3 = 0.017.

#### Mendelian randomization

Two-sample Mendelian randomization (MR) was performed for each exposure and outcome pair using inverse variance weighted (IVW) regression. The significance level was corrected for multiple testing (0.05/6 = 0.008). MR analysis was conducted via the R package TwoSampleMR [46]. Extended documentation on the package can be found at: https://mrcieu.github.io/TwoSampleMR/articles/introduction.html; https://mrcieu.github.io/TwoSampleMR/. MR Egger regression was used to detect violations of the instrumental variable assumptions, most notably, assumption 2 mentioned above. Egger regression can provide an effect estimate which is not subject to the violations of the instrumental variable assumptions [47]. To infer the direction of causal associations between the genetic variants (g) and exposure(x) and outcome (y) MR Steiger’s *Z*-test was performed [48]. Inference of directionality can be achieved by testing the difference in absolute correlation between the g-x and g-y. The Steiger test presupposes that the two variables have a causal link and that the SNP is a viable instrument for at least one of them [48]. Rsq were generated for each SNP and trait via linear regression within the UKB.

## Results

### Evidence of colocalization at 146 loci T2D loci

**Fig. 1:**
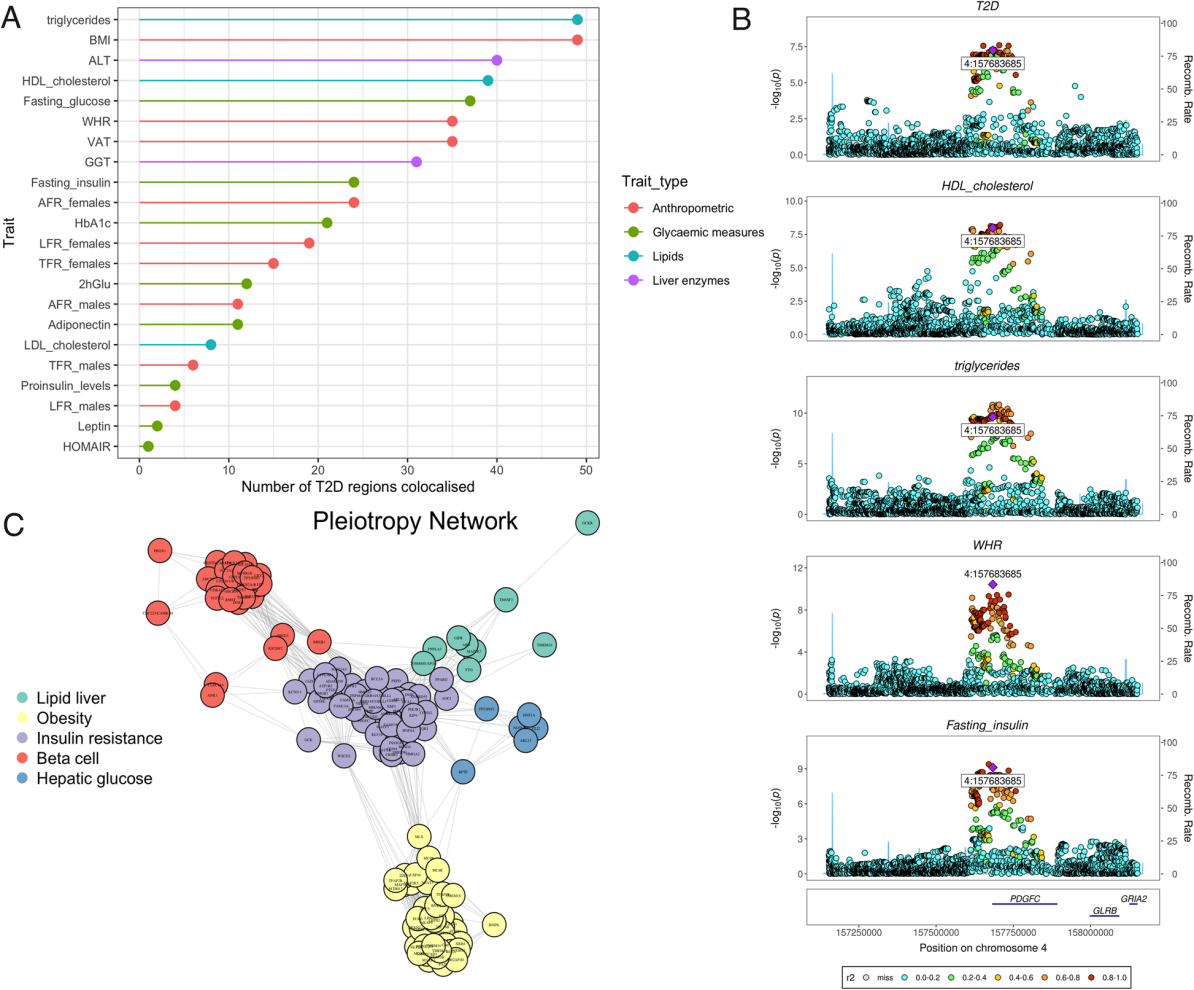
Genome-wide multi-trait colocalization analysis of T2D and 20 related traits. **A** Summary of the number of regions across the genome in which T2D colocalizes with at least one related trait. Traits are labelled by overarching phenotype family, e.g., lipids. **B** A stacked locus plot with an example of the colocalized genetic variant (4:157683685) across T2D, high-density lipoprotein (HDL) cholesterol, triglycerides, waist-to-hip ratio (WHR), and fasting insulin at the PDGFC locus. **C** Network analysis of T2D genetic variants. Variants were clustered according to their pleiotropic associations with related traits plotted into the network, with nodes representing SNPs and the edges the correlations between SNPs based on trait *Z*-scores. SNPs that shared similar associations with metabolic traits clustered together. Five clusters were identified relating to insulin resistance, beta-cell deficiency, obesity, hepatic glucose metabolism, and liver and lipid metabolism. List of abbreviations: body mass index (BMI), alanine transaminase (ALT), high-density lipoprotein (HDL), waist-to-hip ratio (WHR), visceral adipose tissue, gamma-glutamyl Transferase (GGT), arm fat ratio, hemoglobin A_1C_, leg fat ratio, trunk fat ratio, 2-h glucose tolerance test (2hGlu), low-density lipoprotein (LDL), Homeostatic Model Assessment for Insulin Resistance (HOMA-IR)

In order to gain inferences of shared genetic variants across traits, we performed colocalization between T2D and 20 selected metabolic trait loci (Additional file 1: Table S2). Across 243 loci containing T2D genetic risk variants associated at a genome-wide significance level, we identified 146 loci that colocalized with at least one of the 20 selected metabolic trait loci (posterior probability of hypothesis 4 (PP.H4) > 0.6 and GWAS p < 1 × 10^−5^) (Additional file 1: Table S3 and Table S4). BMI and triglycerides colocalized across the most T2D regions with 49 regions each (Fig. 1A), followed by alanine transaminase (ALT) (40 regions), high-density lipoprotein (HDL) cholesterol (39 regions), and fasting glucose (38 regions). We observed evidence of a high degree of pleiotropy across most sites, with 67% of regions (98/146) colocalizing across more than one other trait. The *ANKRD55* (5:55308475–56308475) locus was the most pleiotropic, colocalizing across 13 metabolic traits. Colocalized traits at this locus included lipids (triglycerides, HDL cholesterol), glycemic measures (fasting insulin, fasting glucose), adiposity measures (waist-to-hip ratio, visceral adipose tissue, trunk fat ratio, leg fat ratio, arm fat ratio), and liver enzymes (gamma-glutamyl transferase (GGT), ALT). At the PDGFC locus, colocalization was observed with HDL cholesterol, triglycerides, waist-to-hip ratio, and fasting insulin (Fig. 1B). The presence of these traits collectively indicates a potential association with lipodystrophic insulin resistance.

### Identifying biologically meaningful clusters from pleiotropic associations

To gain insight into the shared biology between T2D genetic risk variants, we extracted variant-trait associations from the summary statistics of traits at colocalized loci. Network-based clustering was performed on a matrix of variant-trait associations (Fig. 1C) to identify groups of T2D genetic risk variants with similar effects across intermediary traits. A network of 1761 edges and 143 nodes was constructed. Next, hierarchical clustering was performed using a spinglass algorithm to detect clusters within the network. In an unsupervised approach, the algorithm determines the optimal number of clusters (*k*). *K* = 10 was determined to be optimal, with a modularity score of 0.66. However, 5 clusters contained fewer than 4 variants making pathway interpretation difficult and thus were removed (see Additional file 3). This left 135 out of 143 SNPs that grouped into 5 clusters (Fig. 1C). The variant-trait associations of each community were assessed using heatmaps (Additional file 1: Table S5 and S6) to discern the key underlying phenotypes driving the grouping of variants. In addition, information about the known biology of the closest genes to variants was also used. The clusters corresponded to the following pathways: (1) obesity, (2) lipodystrophic-like insulin resistance, (3) liver enzymes and lipid metabolism, (4) hepatic glucose metabolism, and (5) beta-cell dysfunction. The obesity cluster contained 40 genetic variants and was defined by positive effects on exclusively BMI, visceral adipose tissue, and arm fat ratio (Additional file 1: Table S6). These variants mapped to well-established obesity-related genes and loci, such as *POC5*, *MC4R*, *TMEM18*, and the *FTO* locus [49–51]. The lipodystrophic-like insulin resistance cluster consisted of 52 genetic variants. This cluster was defined by positive effects on fasting insulin levels, waist-to-hip ratio, and triglycerides; yet, there were negative effects on BMI, fat ratio phenotypes, adiponectin, and HDL cholesterol (Additional file 1: Table S6). Variants within this cluster mapped to genes with known effects on insulin resistance, including *IGF2*, *INSR*, *KLF14* [52], and *PPARG* in adipose tissue [7, 53]. Enriched GO biological processes included phosphatidylinositol 3-kinase signaling, glucose homeostasis, and cellular response to peptide hormone stimulus. The liver and lipid metabolism cluster included 9 genetic variants and was defined by unique associations with three key traits: increased ALT, decreased triglycerides, and decreased low-density lipoprotein (LDL) cholesterol levels (Additional file 1: Table S6). In addition, variants within this cluster mapped to known risk genes influencing liver and lipid metabolism, including *GCKR*, *PNPLA3*, *TM6SF2*, and *APOE* [54–57]. Enriched GO pathways included lipid homeostasis, triglyceride homeostasis, and acylglycerol homeostasis. The hepatic glucose metabolism cluster contained 6 variants and was defined by positive associations with gamma-glutamyl transferase (GGT) (Additional file 1: Table S6). These variants mapped to genes related to hepatic glucose metabolism. *HNF1A* regulates the expression of *SLC2A2* (or *GLUT2*) in the liver [58]. *NDUFS4* and *NDUFAF6* encode nuclear-encoded accessory subunits of the mitochondrial membrane respiratory chain NADH dehydrogenase (complex I). The inhibitory effect of the metformin drug on the mitochondrial respiratory chain Complex I is one of the leading molecular mechanisms of the drug mechanism of action [59]. The beta-cell deficiency cluster featured 28 genetic variants and was defined by positive effects on exclusively glucose-related traits, including fasting glucose, HbA_1C_, 2-h glucose tolerance tests, and proinsulin levels (Additional file 1: Table S6). Variants within this cluster mapped to T2D risk genes influence insulin secretion, including *TCF7L2*, *SLC30A8*, *MTNR1B*, *ADCY5*, and *CAMKD1* [60–64]. GO pathways include the regulation of insulin secretion and glucose homeostasis.

### Pathway polygenic risk scores were associated with anticipated metabolic measures

**Fig. 2:**
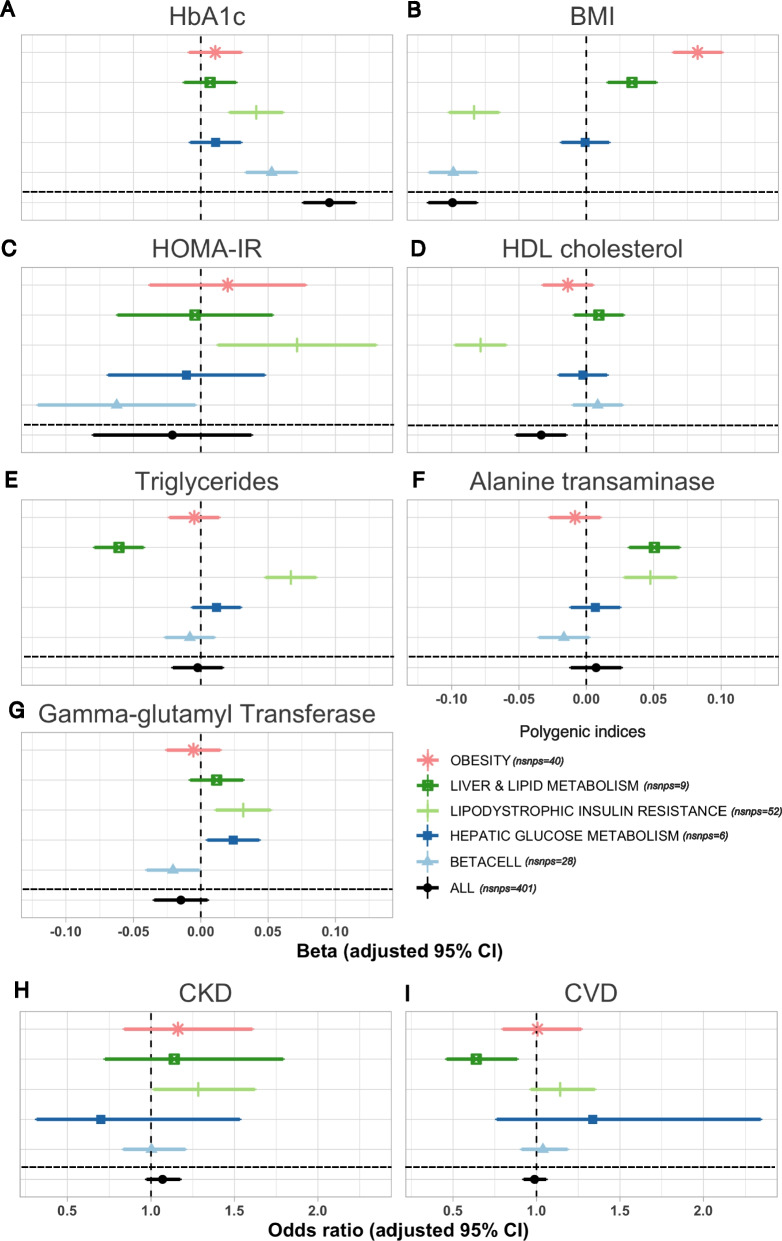
Forest plots of the associations of pathway PRSs with metabolic measures in individuals with type 2 diabetes from three cohorts. **A** HbA_1C_ (*n* = 18,517). **B** BMI (*n* = 21,281). **C** HOMA-IR (Homeostatic Model Assessment for Insulin Resistance) (*n* = 2241). **D** HDL cholesterol (*n* = 19,370). **E** Triglycerides (*n* = 20,797). **F** Alanine transaminase (*n* = 19,134). **G** gamma-glutamyl transferase (*n* = 16,900). **H** Chronic kidney disease (*n* = 19,171). **I** Cardiovascular disease (*n* = 20,504). Linear regression was conducted for continuous outcomes (**A,C,D,E,F,G**) and logistic regression for binary (**H,I**) controlling for age, sex, BMI, and cohort, besides BMI (**B**) which was controlled for sex and cohort. See Additional file 1: Table S8 for the numbers underlying this figure

PRSs were constructed using T2D genetic risk variants for each cluster and tested against relevant metabolic measures for 21,742 individuals with T2D across three cohorts (Additional file 1: Table S7). PRSs were tested for associations with BMI, HbA_1C_, Homeostatic Model Assessment for Insulin Resistance (HOMA-IR), triglycerides, HDL cholesterol, alanine transaminase (ALT), and gamma-glutamyl transferase (GGT). Principally, we observed that individuals with a high genetic burden of a particular T2D risk pathway have distinct clinical characteristics expected for that pathway (Fig. 2A–G, Additional file 1: Table S8). For instance, a high genetic burden for lipodystrophic insulin resistance (Beta 0.04 SD, 95% CI 0.02–0.06, *p* = 2.82 × 10^−07^) or beta-cell dysfunction (Beta 0.05 SD, 95% CI 0.03–0.07, *p* = 2.32 × 10^−12^) was associated with high HbA_1C_ possibly reflecting worse glycemic control in individuals with T2D (Fig. 2A). However, variants within the lipodystrophic-like insulin resistance cluster were associated with high HOMA-IR (Beta 0.07 SD, 95% CI 0.01–0.13, *p* = 8.12 × 10^−03^) and beta-cell deficiency cluster to low HOMA-IR (Beta − 0.06 SD, 95% CI [− 0.12, − 0.01], *p* = 2.85 × 10^−02^) (Fig. 2B), whereas a high-obesity pathway genetic burden did not present significant differences in HbA_1C_ levels but displayed higher BMI (Beta 0.08 SD, 95% CI 0.06–0.10, *p* = 8.0 × 10^−33^) (Fig. 2B). Furthermore, individuals with a high genetic risk in the lipodystrophic insulin resistance pathway were strongly associated with adverse metabolic features of increased triglycerides and decreased HDL cholesterol and BMI (Fig. 2C–E). Finally, individuals with T2D and a high liver and lipid metabolism PRSs consistently had increased BMI and ALT (Fig. 2F), whereas the individuals with the highest hepatic glucose metabolism cluster PRS mostly had higher GGT and triglycerides. For comparison, a PRS was constructed from all T2D genetic risk variants before colocalization (401 SNPs). Pathways often displayed distinct associations with those observed for the overall T2D genetic risk. Interestingly, in some instances the overall T2D risk effect on a trait was nullified, in some instances, due to opposing effects across different genetic pathways, as is the case with triglycerides. Associations between PRSs and metabolic measures remained significant after adjusting for glucose and/or lipid-lowering medications (Additional file 1: Table S9). Comparing individuals in the 90th to 10th percentile of PRSs resulted in similar associations across the entire PRS distribution but with larger effect sizes (Additional file 1: Table S10).

### Determining the directionality and causality between adiposity and T2D

**Fig. 3:**
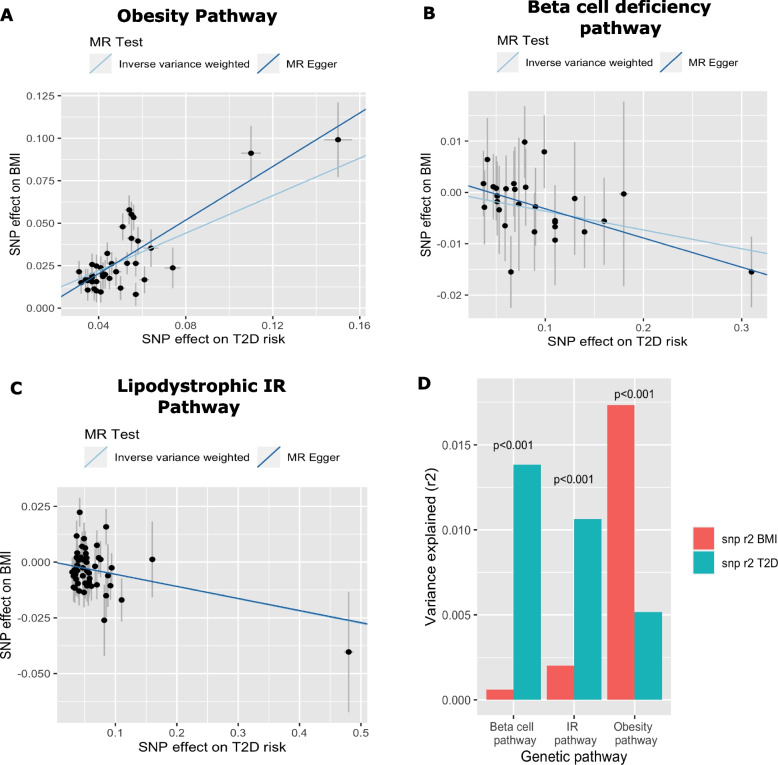
We assessed the causal association between BMI and T2D by comparing the effect sizes of genetic variants mapping to insulin resistance, beta-cell deficiency, and obesity pathway. Regression lines represent causal estimates from Mendelian randomization (MR) methods inverse variance weighted (IVW) regression and Egger regression. Lines represent one standard error. **A** The effect of genetic variants within the obesity pathway with BMI as exposure and T2D as outcome. **B** The effect of variants within the IR (insulin resistance) pathway with T2D as exposure and BMI as outcome. **C** The effect of variants within the beta-cell deficiency pathway with T2D as exposure and BMI as outcome. **D** Bar plots depicting *r*-squared of genetic variants on both BMI and T2D with the resulting Steiger *Z*-test *p*-values

Given the observed heterogeneity in effect estimates, we assessed whether a causal relationship exists in the association between T2D risk pathways scores and BMI. We assessed the evidence for causality using two approaches. First, we compared the correlation between variant effect sizes for T2D and BMI. Correlated proportional increases between the effect sizes of both traits would suggest evidence for a causal association. We extracted T2D genetic risk variants mapping to the obesity, beta-cell deficiency, and insulin resistance pathways from BMI summary statistics. Primarily, we noticed that T2D genetic risk variants mapping to the obesity pathway had consistent, correlated effects with BMI (Fig. 3A). We observed a dose–response relationship between the effects (Pearson *r* = 0.86). However, T2D genetic risk variants mapping to the insulin resistance and beta-cell pathways overall did not have correlated effects (Fig. 3B, C). Next, we assessed causality using an MR Steiger approach to obtain a causal effect estimate for each T2D risk pathway and BMI while also assessing the directionality of the association. In summary, we observed a significant causal effect between increased T2D risk and increased BMI for genetic variants within the obesity pathway (IVW Beta 0.55, 95% CI 0.45–66, *p* = 8.01 × 10^−44^). Egger regression displayed a consistent causal estimate (Egger Beta 0.79, 95% CI 0.39–1.19, *p* = 3.12 × 10^−05^) independent of horizontal pleiotropy. Variants with the obesity pathway explained more variance of BMI (*r*2 = 0.02) than T2D (*r*2 = 0.001). Steiger *Z*-test indicated that the causal direction of the association was in the expected direction with BMI affecting T2D risk (*Z*-test, *p* < 1 × 10^−200^) (Fig. 3D). Evidence for a negative effect between T2D and BMI was observed for both beta-cell (IVW Beta − 0.04, 95% CI − 0.06: − 0.01, *p* = 2.06 × 10^−02^) and insulin resistance pathways (IVW Beta − 0.05, 95% CI − 0.09: − 0.01, *p* = 1.52 × 10^−02^), with no evidence of horizontal pleiotropy (Fig. 3D) (Additional file 1: Table S11 and S12). However, the results of the Steiger *Z*-tests suggested a reverse causal relationship, indicating that T2D may influence BMI, contrary to our initial expectation of BMI affecting T2D risk (Fig. 3D) (Additional file 1: Table S11 and S12). We observed stronger evidence for a causal effect of visceral adiposity on T2D using genetic variants within the obesity pathway (IVW Beta 0.53, 95% CI 0.45–0.60, *p* = 1.89 × 10^−71^, Egger: Beta 0.69, 95% CI 0.40–98, *p* = 6.81 × 10^−07^) than that of BMI, indicating that increased visceral adiposity is driving T2D risk within this pathway.

### Children with a high-obesity-mediated T2D risk have increased adiposity

To further assess directionality, evidence of causality, and the magnitude of effect at different life stages, we tested whether the obesity, lipodystrophic insulin resistance, and beta-cell pathway PRSs were associated with BMI and fat mass percentage in children aged between 9 and 12 years old. PRSs were generated in 3866 children from the Generation R (GenR) Study. Children with a high genetic burden of the obesity pathway had increased BMI (Beta 0.55 SDS, 95% CI 0.35–0.77, *p* = 4.3 × 10^−10^) and had increased fat mass percentage (Beta 0.03, 95% CI 0.02–0.04, *p* = 3.9 × 10^−07^) in comparison to those with a low genetic burden. However, we observed no significant association with BMI, or fat mass percent for the lipodystrophic insulin resistance and beta-cell pathway PRSs (Additional file 1: Table S13).

### The association of polygenic risk scores to clinical outcomes

Lastly, to assess whether partitioned PRSs were associated to hard clinical outcomes, we tested their association to chronic kidney disease (CKD) and cardiovascular disease (CVD). Across the three cohorts, 2559 individuals were diagnosed with CKD and 6357 with CVD.

The lipid and liver metabolism PRS was significantly associated with lower odds of CVD (OR 0.64, 95% CI 0.46–0.88, *p* = 0.002). The lipodystrophic insulin resistance PRS was significantly associated with higher odds of CKD (OR 1.29, 95% CI 1.02–1.62, *p* = 0.03). No other PRSs were significantly associated to CVD or CKD, including the unpartitioned overall PRS (Fig. 2G, I, Additional file 1: Table S14). Results remained consistent after adjustment for lipid-lowering and anti-diabetic medication (Additional file 1: Table S15).

## Discussion

In this study, we assessed the genetic overlap between T2D and 20 intermediary metabolic traits. Our analyses inferred evidence of colocalization across 146 out of 243 T2D loci. We identified five clusters representing T2D pathways, corresponding to beta-cell deficiency, lipodystrophic-like insulin resistance, obesity-mediated, hepatic glucose metabolism, and liver and lipid metabolism. Our findings are consistent with previous studies, highlighting the effectiveness of colocalization in generating meaningful clusters and providing a reliable assessment of pleiotropy [4, 9–11]. We observed distinct degrees of heterogeneity in the effect estimates with metabolic measures across the different T2D pathways, as well as, clinical outcomes such as CVD and CKD. The observed heterogeneity in effects across PRSs and metabolic measures demonstrates the efficacy of partitioning of genetic variants to effectively categorize individuals with distinct clinical characteristics that likely contribute to their type 2 diabetes and distinct clinical complications. Identifying these phenotypically diverse sub-groups of T2D became possible only after stratifying the overall T2D PRS. This highlights the potential pitfalls in applying unstratified PRSs for preventative strategies within heterogeneous diseases [65]. Our study distinguishes itself from previous research by utilizing colocalization to obtain inferences of shared causal variants across GWAS before clustering variants. Moreover, we expanded upon earlier methodologies by assessing the causality and directionality of specific clusters.

We compared our clusters to those generated by Kim et al. [10] and observed broadly similar associations to metabolic measurements (Additional file 3). In relation to clinical outcomes the lipodystrophic PRS was associated to an increased in the odds of CKD in our cluster but not in the Kim et al. lipodystrophic cluster. In summary, the colocalization first approach is a more stringent approach, which boasts the additional advantage: the capability to infer shared causal variants across GWAS. However, the approach of Kim et al. is more computationally trackable with a larger number of GWAS and produces similar associations.

An interesting finding was the heterogeneity in the associations between PRSs and BMI. We observed associations between PRSs and lower BMI for the lipodystrophy, beta-cell, and overall clusters. It is essential to consider that the negative associations with BMI are relative to other individuals with diabetes in our study cohort. Consequently, the observed negative association between T2D PRS and BMI could be explained by the fact that individuals with a higher genetic predisposition to T2D may have a lower proportion of their T2D risk attributed to environmental factors, such as increased BMI.

Despite this causality and directionality analyses also revealed that individuals with a high genetic risk for type 2 diabetes (T2D) within the lipodystrophic insulin resistance or beta-cell pathway were paradoxically associated with lower body mass index (BMI). We validated these causal inferences by applying the PRSs to a pediatric cohort with the reasoning that in these individuals the variants had not yet incremented the risk of T2D, because T2D typically becomes overt in adulthood. If our causal inferences held, we expected to observe increased adiposity for those children with a high-obesity pathway genetic risk, since adiposity directionally effects T2D. On the other hand, for the lipodystrophic insulin resistance and beta-cell pathways, we did not expect to observe any differences in BMI, as T2D risk variants have not exerted any effect at such an earlier life stage. Indeed, this is exactly what we observe. These observations are consistent with the hypothesis that BMI causally influences T2D risk through the obesity pathway but not the beta-cell and insulin resistance pathways. This finding is supported by the similar power and effect sizes of the PRSs in the adult cohort populations with regard to BMI (F-statistics: 49–55). We demonstrated the potential of utilizing pediatric cohorts for determining the direction of genetic effects across different traits. The connection between increased T2D risk and lower BMI might be influenced by worse glycemic control, as evidenced by the PRSs that shows the most robust association with both lower BMI and higher HbA_1C_. Possible explanations for this weight loss in relation to worse glycemic control could be other disease complications, such as glycosuria [66] or muscle wasting [67].

From these findings, we hypothesize that individuals with high genetic risk in the lipodystrophic insulin resistance and beta-cell pathways may not experience significant benefits from weight loss interventions due to their already lower BMI compared to other individuals with diabetes. Although weight gain, in these leaner individuals at higher risk of T2D, may still exacerbate the dysfunctional glycemic pathway, treatments focusing on glycemic control rather than weight loss-specific interventions may be more beneficial for individuals with high genetic risk in these pathways. Additionally, the relationship between decreased BMI in these sub-groups provides support for the lean diabetes phenomena often observed in certain ethnic groups [68]. Nevertheless, clinical trials will be necessary to see if weight loss may still have a certain extent of insulin sensitizing effect which could improve glycemia or not.

Curiously, genetic variants associated with T2D comprised by the obesity pathway conferred a relatively “healthy” metabolic profile, as they lack significant associations with other metabolic traits such as HbA_1C_, lipid levels and liver function markers, or HOMA-IR and show no association to higher odds of CVD or CKD. Therefore, these individuals could be considered as “metabolically healthy obese” as previously characterized [69]. However, they do not precisely correspond to the conventional notion of “metabolically healthy obese” individuals, as they are associated with an elevated risk of T2D and therefore cannot be considered “healthy.” It seems that these variants are connected to increased risks for both T2D and obesity, operating independently of traditional metabolic pathways. Exploring the functions of the variants within this pathway could provide insights into the mechanism by which these individuals remain relatively healthier.

Clusters containing a small number of variants present a challenge since they do not contain enough trait associations to make inferences of the pathway they are acting on. To address this, we excluded clusters containing three or less variants from our main analysis. To avoid selection bias, we tested these cluster in the same manner as in the main results. We observed distinct heterogeneity in three of the clusters. However, these clusters contained only one variant; consequently, interpretation of the pathway for these clusters was not possible. PRSs of the remaining two clusters showed no evidence of an association to metabolic measures. Clustering can also be impacted by the intermediary GWAS traits selected. In this specific instance, the selected traits were more heavily weighted toward characteristics associated with insulin resistance. Thus, the cluster containing the largest number of genetic variants was the lipodystrophic cluster. We conducted sensitivity analysis to assess how clustering changed when an insulin secretion phenotype, oral glucose tolerance test (OGTT) of low sample size (*n* = 5318) was included (see Additional file 3). We observe that the addition of OGTT can affected the clustering of variants. However, the resolution for distinguishing other clusters was diminished as a consequence of including a GWAS with so few variants associated.

It is important to highlight that in certain aspects, the PRS generated using all 401 genetic variants demonstrated superior performance compared to the stratified PRSs. The overall PRS exhibited a stronger association with HbA1C compared to any other PRS. However, the use of the stratified PRS allows the elucidation of the underlying mechanisms of associations, which is lacking in the unstratified PRS. For instance, the beta-cell or insulin resistance PRSs revealed links to low and high HOMA-IR levels, respectively. This suggests that higher HbA1C levels are influenced by both insufficient insulin production and insulin resistance. These findings suggest that while a comprehensive score might yield a more potent prediction of HbA1C levels, the partitioned scores provide nuanced etiological insights that the comprehensive PRS score may not fully encapsulate.

Our study is not free of limitations. First, not all clinical variables were available for all the participants across all cohorts. Notably, HOMA-IR measures were only available for one of the cohorts limiting our power to detect associations in related to this parameter. Second, our inferences of genetic overlap are based solely on loci with detectable variants (*p* < 1 ×  − 10^−5^). Third, although only GWAS summary statistics of European ancestry were used in our study, they still have a range of sample sizes and underlying differences in genetic architectures, reducing the probability of observing colocalization. Fourth, previous studies have noted that obtaining LD information from a population different from that used to conduct the GWAS can influence fine-mapping. This can influence the variants identified in credible sets and thus the multi causal variant colocalization results [70]. Fifth, our results can also be biased by the metabolic trait GWAS we selected to test for colocalization. We also opted to use a hard clustering method over a soft one, which does not allow a variant to belong to more than one cluster. Therefore, we may obviate the impact of variants that act on multiple pathways. Additionally, our decision to use a hard clustering approach led to a high number of unassigned variants (*n* = 8). The inability to assign certain variants to any specific cluster may lead to less robust PRSs, which could influence the overall performance of our method. Lastly, while we adjusted for medication use in a sensitivity analysis, glycemic phenotyping of participants with established T2D can be subject to artifacts introduced by pharmacological treatment. We were able to identify a vast number of pleiotropic loci acting across multiple traits. Despite this, we could not find colocalization at 97/243 T2D loci (40%) across any of the 20 metabolic traits tested. This highlights the utility of applying a statistical framework to test the genetic overlap of GWAS signals, considering that we observed no evidence of colocalization across many regions. Attempting to gain inferences from these non-overlapping genetic variants can introduce false positives and erroneous associations. Further, it suggests that many T2D loci are driven by non-common metabolic traits; or, that colocalization methodologies are too conservative; or, that many metabolic GWAS are still underpowered. A hypothesis-free and large-scale approach to testing traits would provide more insights into the biology of each locus; however, at the expense of phenotypic interpretability of the pathways [71]. Further still, a more progressive approach would be to integrate additional intermediary phenotypes across multiple molecular layers, such as mQTLs, pQTLs, and eQTLs to provide more comprehensive biological information [72]. GWAS sample sizes will continue to grow making pleiotropic inferences more common across most loci, consequently reducing the biological interpretability at such loci. Our findings may have implications for individuals with uncontrolled T2D. We might better characterize these persons with T2D to tailor specific treatments. We hypothesize that individuals with T2D and a high-obesity cluster PRS might benefit best from weight loss and medical treatments with concomitant weight loss, such as glucagon-like peptide-1 (GLP-1) receptor agonists or sodium-glucose co-transporter 2 (SGLT2) inhibitors; and those with a high PRS for beta-cell dysfunction may display insulin deficiency and may initially try sulfonylureas derivates or need insulin therapy already early on. While most individuals’ risk will be driven by moderate genetic burden across multiple pathways, those with high risk in a particular pathway could be targeted for more specific treatments. Here we should acknowledge the caveat that people with the same static polygenic score for a certain T2D subtype might find themselves in different metabolic states depending on age, stage in disease progression, environmental factors, treatment history, etc. This could be evaluated in a randomized control trial in which tailored treatments are applied to individuals in the highest risk of a particular partitioned PRS.

## Conclusions

In summary, we successfully applied colocalization and network clustering analyses to the heterogeneous and polygenic complex disease of T2D. Using a colocalization first approach allowed us to infer shared causal variants across multiple GWAS. Partitioned PRSs were associated to unique metabolic and clinical outcomes indicating successful partitioning of heterogeneity. Directionality and causality analyses allowed us to infer evidence of an association between T2D risk on lower BMI for lipodystrophic insulin resistance and beta-cell dysfunction pathways. Our work expands on previous approaches by providing stronger inferences of pleiotropy, causality, and directionality of GWAS variant-trait associations. We infer biologically meaningful interpretations of pleiotropic genomic loci and demonstrate the potential of partitioned PRSs for personalized medicine.

### Supplementary Information

**Additional file 1: Table S1.** Cohort descriptives and measurements. **Table S2.** List of GWAS summary statistics used and features of the studies. **Table S3.** Results of Hyprcoloc colocalization. **Table S4.** Results of SuSiE colocalization. **Table S5.** SNP correlation matrix used to construct network. **Table S6.** Variant-trait z-scores for clusters. **Table S7.** SNPs used to construct PGIs. **Table S8.** Associations between PGIs and metabolic outcomes. **Table S9.** Associations between PGIs and metabolic outcomes controlling for medication usage. **Table S10.** Associations of individuals in top and bottom deciles to metabolic outcomes. **Table S11.** Results of Mendelian randomization between BMI/VAT and clusters. **Table S12.** Results of Steiger z test. **Table S13.** Associations of PGIs in GENR pediatric cohort. **Table S14.** Associations of PGIs and clinical outcomes. **Table S15.** Associations of PGIs and clinical outcomes adjusting for medication. **Table S16.** Associations between PGIs and metabolic outcomes for clusters with fewer than 4 variants. **Table S17.** Associations between PGIs and metabolic outcomes for Kim et al. clusters.

**Additional file 2.** Supplementary methods file. Provides additional details of cohort measurements and statistical methods are presented under the following sub-headings: UK Biobank measurements, Rotterdam Study measurements, The DiaGene Study measurements, The Generation R Study, Colocalization, Clustering, PRS, and Mendelian Randomization.

**Additional file 3.** Supplementary information. Results and figures relating to additional analyses conducted are presented under the following sub-headings: Assessment of clusters with fewer than 4 variants, Comparison with clusters generated by Kim et al., and Sensitivity analysis.

## Data Availability

This research has been conducted using the UK Biobank Resource (accession ID: 67,864). Data is available for bona fide researchers from the UKBB Resource [http://www.ukbiobank.ac.uk/about-biobank-uk/], on filing an application. T2D GWAS summary statistics can be downloaded from DIAGRAM consortium website [https://diagram-consortium.org/downloads.html] [4]. ALT and GGT GWAS summary statistics can be downloaded from [https://www.ebi.ac.uk/gwas/downloads/summary-statistics] with the accession numbers GCST90013405 and GCST90013407 [26]. Triglycerides, HDL, and LDL cholesterol GWAS summary statistics can be downloaded from [https://gwas.mrcieu.ac.uk] using the following IDs: ieu-b-111, ieu-b-109, and ieu-b-110 [27]. VAT GWAS summary statistics can be downloaded from [https://www.ebi.ac.uk/gwas/downloads/summary-statistics] with the accession number GCST008744 [28]. Fat ratio phenotype GWAS summary statistics can be downloaded from [https://myfiles.uu.se/ssf/s/readFile/share/3993/1270878243748486898/publicLink/GWAS_summary_stats_ratios.zip] [29]. BMI and WHR GWAS summary statistic data can be downloaded from [https://doi.org/10.5281/zenodo.1251813] [30]. Fasting proinsulin, HOMA-IR, Insulin Sensitivity Index, fasting glucose, 2 h-glucose test, HbA1c, and fasting insulin GWAS data can be downloaded from [https://magicinvestigators.org/downloads/] [31–33, 36]. Leptin GWAS summary statistics can be downloaded from [https://www.ebi.ac.uk/gwas/downloads/summary-statistics] with accession number GCST003367 [34]. Adiponectin GWAS summary statistics can be downloaded from [https://www.ebi.ac.uk/gwas/downloads/summary-statistics] with accession number GCST001465. All data generated or analyzed during this study are included in this published article and its supplementary information files.

## Abbreviations

GWAS: Genome-wide association study
LD: Linkage disequilibrium
T2D: Type 2 diabetes
PRSs: Polygenic risk scores
MR: Mendelian randomization
SNP: Single-nucleotide polymorphism
BMI: Body mass index
ALT: Alanine transaminase
HDL: High-density lipoprotein
WHR: Waist-to-hip ratio
GGT: Gamma-glutamyl transferase
2hGlu: 2-h glucose tolerance test
HbA_1c_: Hemoglobin A_1C_
LDL: Low-density lipoprotein
HOMA-IR: Homeostatic Model Assessment for Insulin Resistance
