# Supplementary material for: Defining type 2 diabetes polygenic risk scores through colocalization and network-based clustering of metabolic trait genetic associations
Source: Genome Med. 2024 Jan 10;16:10. doi: 10.1186/s13073-023-01255-7 (PMC10777532; doi:10.1186/s13073-023-01255-7)
Supplement: Supplementary file 2 — Additional file 2. Supplementary methods file. Provides additional details of cohort measurements and statistical methods are presented under the following sub-headings: UK Biobank measurements, Rotterdam Study measurements, The DiaGene Study measurements, The Generation R Study, Colocalization, Clustering, PRS, and Mendelian Randomization. [file 13073_2023_1255_MOESM2_ESM.docx]

Supplementary methods

UK Biobank measurements

In the UK biobank, blood samples were collected in a fasting state at baseline (2006-2010). Biochemistry assay quality procedures and companion documents for serum biomarker data can be found online ([www.biobank.ctsu.ox.ac.uk](http://www.biobank.ctsu.ox.ac.uk)). Briefly, plasma HbA1C levels were measured using variant II turbo Hemoglobin Testing System; from Bio-Rad. LDL was measured via by enzymatic protective selection analysis on a Beckman Coulter AU5800. HDL, by enzyme immuno-inhibition analysis on a Beckman Coulter AU5800. Triglycerides, by GPO-POD analysis on a Beckman Coulter AU5800. ALT, by IFCC analysis on a Beckman Coulter AU5800. GGT, by IFCC analysis on a Beckman Coulter AU5800. Cardiovascular disease was defined using the International Statistical Classification of Diseases and Related Health Problems (ICD) version 10 codes I20, angina pectoris; I21, acute myocardial infarction; 122 subsequent ST elevation (STEMI) and non-ST elevation (NSTEMI) myocardial infarction; I23, certain current complications following ST elevation (STEMI) and non-ST elevation (NSTEMI) myocardial infarction; I24, other acute ischaemic heart diseases; or I25, chronic ischaemic heart disease. Chronic kidney disease was defined by ICD-10 code N18; Chronic kidney disease. Medication data was self-reported by individuals during a verbal interview at the assessment centre. Anti-diabetic medication consisted of; “insulin product”, “metformin”, “glibornuride”, "glibenclamide”, “daonil”, “diabetamide”, “gliclazide”, “glipizide product”, “gliquidone”, “glimepiride”, and “gliclazide”. Lipid lowering medication consisted of; “atorvastatin”, “fluvastatin”, “pravastatin”, “rosuvastatin”, “simvastatin”.

Rotterdam Study measurements

In the Rotterdam study, blood samples were collected in a fasting state across multiple cohorts and visits. Data was collected from all available cohorts and visits. In 1990, non-fasting serum total cholesterol and HDL-cholesterol levels were measured using enzymatic colorimetric methods (Kone Specific Analyzer, Kone Instruments). Starting from 1997, fasting total cholesterol, HDL-cholesterol, and fasting triglyceride levels were determined using similar enzymatic procedures (Hitachi Analyzer, Roche Diagnostics). Non-HDL-cholesterol was calculated by subtracting HDL-cholesterol from total cholesterol. The Friedewald equation was employed to estimate LDL-cholesterol levels. Glucose and alanine aminotransferase (ALT), were assessed by automatic enzyme procedures and insulin with automatic immunoassay (Roche Diagnostics). HOMA‐IR was calculated using a computer model with glucose (mmol/L) and insulin (mmol/L) [1]. Cardiovascular disease encompassed instances of myocardial infarction (MI), coronary heart disease and occurrences of strokes. Information pertaining to these events was garnered through digital linkage with records held by general practitioners, as well as through reviews of discharge reports furnished by medical specialists. The criteria used to define mortality stemming from coronary heart disease were grounded in the codes provided by the ICD-10. Medication data was self-reported by individuals during a verbal interview at the assessment centre. Individuals indicated the medication they had taken in the past week. Medications were then grouped by research assistants. The ‘anti-diabetic therapy’ and ‘Serum reducing agents’ groupings were used.

The DiaGene Study measurements

In the DiaGene Study, data on anthropometrics and fasting blood measures was obtained through patient medical records 6 months prior to or after inclusion [2]. Cardiovascular disease was comprised of myocardial infarction, percutaneous coronary intervention/coronary arterial bypass graft (PCI/CABG), cerebrovascular accident, transient ischemic attack, and peripheral arterial disease defined by patient medical records. Chronic kidney disease was also obtained through patient medical records. Medication data was self-reported by individuals during via questionnaire. Medication consisted of; “insulins and analogues”, “metformin”, and “sulfonylurea derivatives”.

The Generation R Study

Within the Generation R study, fat percentage was calculated via Dual-energy- X-ray absorptiometry (DXA). DXA can quantify fat mass and a percentage via low X-ray exposures (5-10 mirco Sv). DXAs were performed using an iDXA densitometer. Total body scans were used to derive total body fat percentage. BMI was calculated weight (kg)/ height (m^2^) and was then transformed into age and sex standard deviation scores (SDS) using LMSGrowth [3].

Colocalization

We sought to use colocalization to gain inference of pleiotropic associations between GWAS variants. If the pleiotropy is directional, such that the effect of the genetic variant on the outcome occurs through the intermediary phenotype (g => x => y), then the intermediary phenotype can be said to be "mediating" the genetic signals of the outcome [4]. We refer to this phenomenon as mediated pleiotropy. Determining whether variants act through intermediary traits can help characterize the underlying pathways through which these variants exert their effects. However, the presence of linkage disequilibrium (LD) between putative causal and non-causal/marker variants makes it challenging to assess the overlap of GWAS signals between traits. For instance, a specific marker can be associated at the genome-wide significant level with two independent traits while actually being in LD with distinct causal variants. Additionally, the marker can be located at a considerable distance (e.g., hundreds of kilobase pairs) from the true causal variant. This issue is further exacerbated by the high polygenicity of complex traits, as revealed by the increasingly larger sample sizes of GWAS. New methodologies have addressed these limitations by providing formal statistical tests to determine whether two signals from different traits are shared within a locus. This approach is known as colocalization [5-7]. Colocalization enables a robust assessment of the genetic overlap across traits. To fulfill a crucial assumption for colocalization, all GWAS data must be either directly genotyped or well-imputed (r2 > 0.3). The summary statistics for the T2D GWAS were obtained from genotyped data that were previously imputed using the HRC r1.1 reference panel [19]. Consequently, summary statistic data for related traits needed to be imputed to a comparable level of SNP coverage. For GWAS data that were not adequately well-imputed (such as HapMap or 1000G), we conducted summary statistic imputation using the SSIMP software and a subsample of the HRC r1.1 reference panel (n = 27,125) [33]. After performing imputation quality control, only SNPs with a minor allele frequency >1% and r2.pred > 0.3 were retained for further analyses. Mahajan reported 243 loci containing SNPs at genome-wide significance (GWS) with T2D. For colocalization analysis, a region of 1 megabase pair (500 kb on either side) around the lead SNP (as reported by Mahajan) was defined. Summary statistic information for all SNPs within these regions was extracted from the available metabolic trait summary statistics. If a specific defined region contained only one T2D causal SNP (identified through GCTA conditional analysis), the probability of colocalization was assessed using Hyprcoloc across all traits simultaneously [7]. If the posterior probability of colocalization (PRPA) was greater than 0.60 (where PR = PA = 0.78) and the p-value of the SNP-trait association was < 1 x 10-5, the genomic region across traits was considered to have significant evidence of colocalization. A posterior probability threshold of 0.6 was chosen to account for population structure differences between the GWAS and reference populations used for calculating LD matrices. Sensitivity analysis was conducted to assess the robustness of colocalized traits to changes in prior probabilities. In cases of colocalization, Hyprcoloc identifies a candidate SNP that explains the highest proportion of the posterior probability of colocalization. This candidate SNP has a high probability of being the causal SNP or in high LD (r2 = 1) with the causal SNP. Therefore, this candidate SNP was extracted from the colocalized trait summary statistics and aligned with the T2D effect-increasing allele. While Hyprcoloc assumes a single causal variant and may have reduced power in detecting colocalization at loci with multiple causal SNPs, its regional probability (PR) test does not make this same assumption. The PR test can be used as an initial step to check whether two genomic regions share a causal variant in close proximity. Regions with P_R_ > 0.8, including those containing multiple causal variants as reported by Mahajan, and single-variant regions with P_R_ > 0.8 but lacking evidence of colocalization (P_R_P_A_ < 0.60), were further investigated using the Sum of Single Effects (SuSiE) coloc framework [5, 8]. The SuSiE coloc framework enables the consideration of multiple causal variants between two genomic regions of interest. It utilizes the 'sum of single effects' regression model to fine-map genetic signals and generate credible sets for GWAS summary statistics. Subsequently, colocalization can be performed between all possible pairs of signals within each trait's credible sets. In the context of T2D, pairwise colocalization testing was conducted between traits and regions using the SuSiE coloc framework. To distinguish signals, SuSiE requires an LD matrix of the variants being tested. LD matrices were calculated using the HRC1.1 reference panel. Regions were considered to have colocalized if the probability of colocalization (H4) was greater than 0.6, and the SNP-trait association p-value was less than 1 x 10-5. Prior to colocalization, locus plots were generated for the regions and manually examined. Additionally, the alignment of alleles between the summary statistics and reference LD matrix was verified using the RSS model under the null hypothesis with a regularized LD matrix. Expected z-scores were computed and compared against the observed values. In some cases, SuSiE was unable to identify any credible sets for a particular genomic region. In such situations, coloc [6] was applied under the assumption of a single causal variant. In cases of significant colocalization between the T2D region and a metabolomic trait region, the T2D lead colocalized causal variant defined by SuSiE was extracted from the summary statistics of the respective trait GWAS. This approach was adopted since SuSiE and COLOC are conducted in a pairwise manner between each trait and T2D. To aggregate the effect of a genetic variant across multiple traits, a common signal is required across all traits. Additionally, using the same variant helps prevent issues when aligning alleles to the same strand. Subsequently, SNPs were aligned to the effect-increasing allele. Palindromic SNPs with ambiguous allele frequencies (> 0.6 MAF > 0.4) were excluded and replaced with LD proxies (r2 > 0.8).

Clustering

Spinglass is an approach from statistical physics, based on the so-called Potts model. In this model, each particle (i.e. vertex) can be in one of c spin states, and the interactions between the particles (i.e. the edges of the graph) specify which pairs of vertices would prefer to stay in the same spin state and which ones prefer to have different spin states. The model is then simulated for a given number of steps, and the spin states of the particles in the end define the communities. The consequences are as follows: 1) There will never be more than c communities in the end (c was set to 200). 2) There may be less than c communities in the end as some of the spin states may become empty. 3) It is not guaranteed that nodes in completely remote (or disconnected) parts of the networks have different spin states.

PRS

Several considerations were taken into account when deciding which PRS percentiles to compare. The percentiles needed to be extreme enough to avoid containing groups with overlapping PRS point estimates, since recent studies have reported large credible intervals in PRSs [9]. Another consideration was to minimize the number of individuals who fall into the top percentiles of multiple PRSs, as to minimize effects that are caused by correlation between traits. Additionally, the percentiles had to contain a large enough sample size of individuals to be able to reliably detect associations.

Mendelian Randomization

Briefly, IVW-MR uses a univariable model to regress SNP-instrument associations with an outcome on SNP-instrument associations with an exposure, weighted by the inverse of the variance in SNP-outcome associations. The IVW intercept is constrained at zero as a result of MR’s “exclusion restriction assumption”, which states that instruments must only be associated with the outcome via the exposure. For a genetic variant to act as a valid instrumental variable, it has to satisfy the following assumptions: the variants needs to be 1) reliably associated with the exposure; 2) associated with the outcome only through the exposure of interest, 3) conditionally independent of the outcome given the exposure and confounding factors. In addition an MR steiger approach was taken. There are some weaknesses to the Steiger test. First, some amounts of horizontal pleiotropy, in which the SNP influences the outcome via a mechanism other than the exposure, could present issues because this can invalidate the instrument. Second, any differences in measurement error between the exposure and the outcome may lead to erroneous causal direction inference. Third, some unmeasured confounding between the exposure and the outcome could lead to the incorrect causal direction being inferred. However, this third weakness is unlikely when the magnitude of the observational variance explained between the exposure and the outcome is below 0.2.

References

1. Levy, J.C., D.R. Matthews, and M.P. Hermans, *Correct Homeostasis Model Assessment (HOMA) Evaluation Uses the Computer Program.* Diabetes Care, 1998. **21**(12): p. 2191-2192.

2. van Herpt, T.T.W., et al., *Introduction of the DiaGene study: clinical characteristics, pathophysiology and determinants of vascular complications of type 2 diabetes.* Diabetology & Metabolic Syndrome, 2017. **9**(1): p. 47.

3. Cole, T.J., *The LMS method for constructing normalized growth standards.* Eur J Clin Nutr, 1990. **44**(1): p. 45-60.

4. Visscher, P.M. and J. Yang, *A plethora of pleiotropy across complex traits.* Nature Genetics, 2016. **48**(7): p. 707-708.

5. Wallace, C., *A more accurate method for colocalisation analysis allowing for multiple causal variants.* PLOS Genetics, 2021. **17**(9): p. e1009440.

6. Giambartolomei, C., et al., *Bayesian Test for Colocalisation between Pairs of Genetic Association Studies Using Summary Statistics.* PLOS Genetics, 2014. **10**(5): p. e1004383.

7. Foley, C.N., et al., *A fast and efficient colocalization algorithm for identifying shared genetic risk factors across multiple traits.* Nature Communications, 2021. **12**(1): p. 764.

8. Wang, G., et al., *A simple new approach to variable selection in regression, with application to genetic fine mapping.* Journal of the Royal Statistical Society: Series B (Statistical Methodology), 2020. **82**(5): p. 1273-1300.

9. Ding, Y., et al., *Large uncertainty in individual polygenic risk score estimation impacts PRS-based risk stratification.* Nature Genetics, 2022. **54**(1): p. 30-39.
