# Supplementary material for: Defining type 2 diabetes polygenic risk scores through colocalization and network-based clustering of metabolic trait genetic associations
Source: Genome Med. 2024 Jan 10;16:10. doi: 10.1186/s13073-023-01255-7 (PMC10777532; doi:10.1186/s13073-023-01255-7)
Supplement: Supplementary file 3 — Additional file 3. Supplementary information. Results and figures relating to additional analyses conducted are presented under the following sub-headings: Assessment of clusters with fewer than 4 variants, Comparison with clusters generated by Kim et al., and Sensitivity analysis. [file 13073_2023_1255_MOESM3_ESM.docx]

Supplementary note

Assessment of clusters with fewer than 4 variants

Clusters containing a small number of variants present a challenge since they do not contain enough pleiotropic associations to make robust inferences of the pathway they are acting on. To tackle selection bias due to the minimum number of variants used to define a cluster we constructed PGIs for clusters with less than four genetic variants and tested them against the same metabolic traits. Clusters 2 (n=1), C3 (n=1), C7 (n=3), C9 (n=2), and C10 (n=1) contained each fewer than 4 variants (Figure S1).
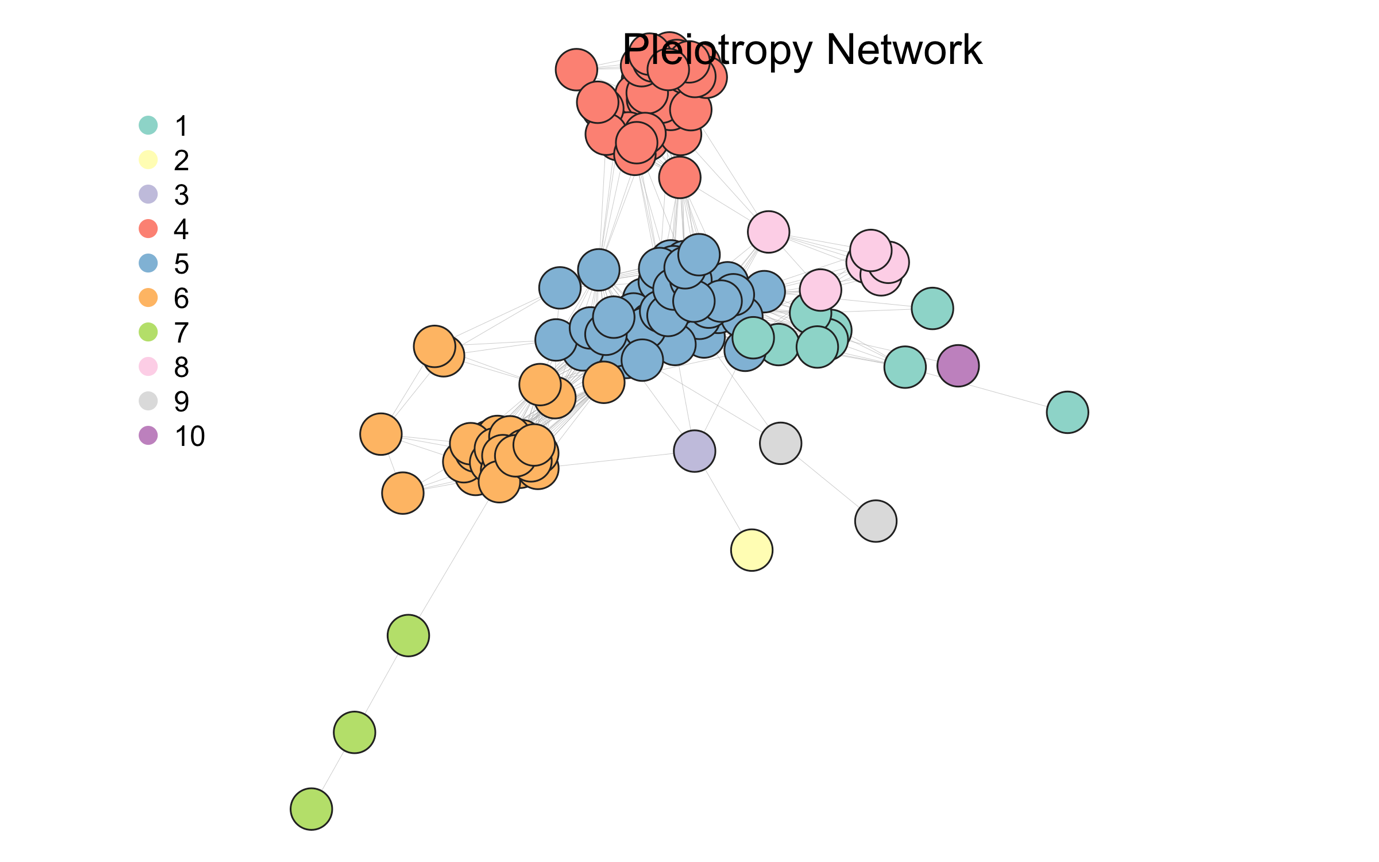


Figure S1 - Network analysis of T2D genetic variants. Variants were clustered according to their pleiotropic associations with related traits plotted into the network, with nodes representing SNPs and the edges the correlations between SNPs based on trait Z-scores. SNPs that shared similar associations with metabolic traits clustered together. Ten clusters were identified. However, clusters C2 (n=1), C3 (n=1), C7 (n=3), C9 (n=2), and C10 (n=1) contained fewer than four variants and thus did not meet the inclusion criteria for this study.

| **SNP** | **Chr** | **Pos** | **Beta** | **SE** | **EA** | **NEA** | **EAF** | **Pvalue** | **gene** | **cluster** |
| --- | --- | --- | --- | --- | --- | --- | --- | --- | --- | --- |
| 6:43811430 | 6 | 43811430 | 0.051 | 0.007 | C | A | 0.29 | 6.20E-13 | VEGFA | 10 |
| 10:114758349 | 10 | 114758349 | 0.31 | 0.0069 | T | C | 0.29 | 0.00E+00 | TCF7L2 | 2 |
| 10:114758349 | 10 | 114758349 | 0.31 | 0.0069 | T | C | 0.29 | 0.00E+00 | TCF7L2 | 2 |
| 5:133864599 | 5 | 133864599 | 0.037 | 0.0064 | A | G | 0.43 | 9.20E-09 | PHF15 | 3 |
| 5:133864599 | 5 | 133864599 | 0.037 | 0.0064 | A | G | 0.43 | 9.20E-09 | PHF15 | 3 |
| 3:124926054 | 3 | 124926054 | 0.037 | 0.0064 | A | G | 0.57 | 7.70E-09 | SLC12A8 | 7 |
| 15:53747228 | 15 | 53747228 | 0.24 | 0.043 | G | C | 0.01 | 2.10E-08 | WDR72 | 7 |
| 11:69463679 | 11 | 69463679 | 0.14 | 0.021 | A | G | 0.97 | 1.10E-10 | CCND1 | 7 |
| 11:69463679 | 11 | 69463679 | 0.14 | 0.021 | A | G | 0.97 | 1.10E-10 | CCND1 | 7 |
| 10:124165615 | 10 | 124165615 | 0.046 | 0.0063 | G | A | 0.52 | 2.50E-13 | PLEKHA1 | 9 |
| 10:124165615 | 10 | 124165615 | 0.046 | 0.0063 | G | A | 0.52 | 2.50E-13 | PLEKHA1 | 9 |
| 19:19460541 | 19 | 19460541 | 0.09 | 0.012 | C | G | 0.071 | 3.00E-13 | TM6SF2 | 9 |

Table S1 – Variant cluster assignments for cluster with n < 4.

Clusters 2, 3, and 10 displayed noteworthy associations with metabolic measures across all three cohorts, as evident from the Figure S2. For instance, cluster 2 showcased links to higher HbA1C levels, along with lower BMI and HOMA-IR index. Notably, these metabolic associations bear resemblance to those observed in the beta-cell cluster. However, it is worth highlighting that the variant within cluster 2 is distinct from those variants within the beta-cell cluster. This indicates that the influence of this variant on insulin secretion likely occurs via a distinct pathway. Cluster 10, on the other hand, exhibited associations with elevated HbA1C, along with reduced HOMA-IR and triglyceride levels. These associations likewise suggest impaired insulin secretion but with the additional involvement of decreased triglyceride levels.

Lastly, cluster 3 displayed an association with lower BMI. Further exploration through additional trait associations is necessary to unveil the mechanisms at play in cluster 3. Notably, clusters 2, 3, and 10 were characterized by a single variant, which complicates the process of drawing definitive interpretations. Conversely, clusters 7 and 9 did not reveal any statistically significant associations with metabolic traits, as depicted in the Figure S2.

The results provided by testing the association of clusters containing a small number of variants underscore the challenges in interpreting such associations. However, we do see evidence that these variants have distinct associations to metabolic outcomes. Including the data from more T2D variants, as well as, more metabolic trait GWAS could improve the interpretability of these clusters.


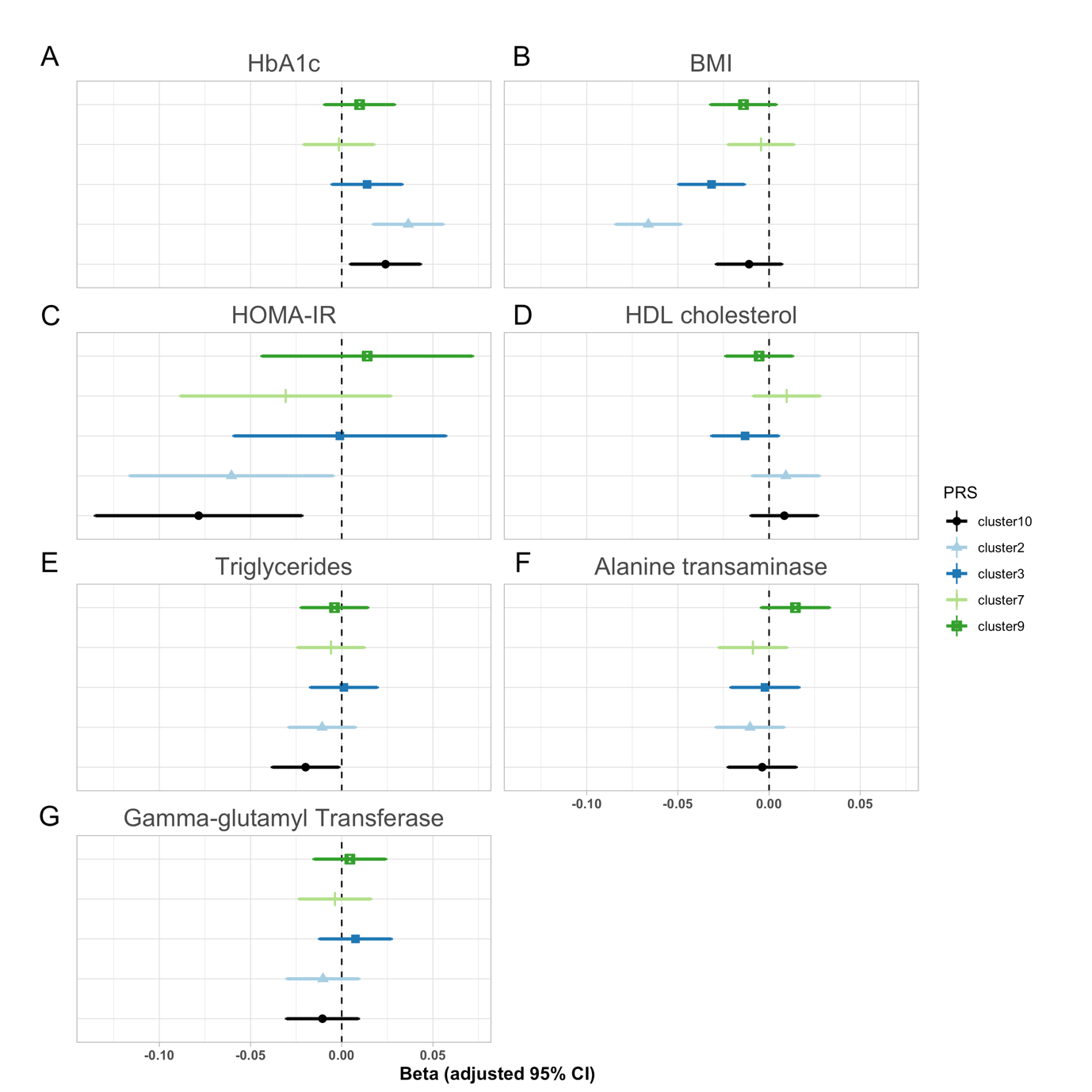


Figure S2 - Forest plots of the associations of pathway PGIs with metabolic measures in individuals with type 2 diabetes from three cohorts. A) HbA1C (n=18,517). B) BMI (n=21,281). C) HOMA-IR (Homeostatic Model Assessment for Insulin Resistance) (n=2,241). D) HDL cholesterol (n=19,370). E) Triglycerides (n=20,797). F) alanine transaminase (n=19,134). G) gamma-glutamyl Transferase (n=16,900). Linear regression were conducted for continuous outcomes (A,C,D,E,F,G) controlling for age, sex, BMI and cohort, besides BMI (B) which was controlled for sex and cohort. See Table S16 for the values underlying this figure.

Comparison with clusters generated by Kim et al

To evaluate the effectiveness of our methodology in comparison to prior clustering approaches, we constructed PGIs using the dataset from the Kim et al 2023 study, focusing on PGIs associated with analogous physiological categories. These encompassed pathways such as the obesity, betacell1, betacell2, lipodystrophy, ALP negative, and liver lipid clusters. Our selection of genetic variants was aligned with clusters contingent on the established weight threshold of 0.832, as delineated in Kim et al.'s findings. We generated corresponding PGIs employing the same source summary statistics from Mahajan et al. while excluding the UK Biobank cohort from the meta-analysis. Subsequently, we tested the associations of these PGIs to the same outcomes as performed in the results presented in the main body of the manuscript. In broad terms, the relationships observed between cluster PGIs and metabolic outcomes exhibit a high degree of consistency between the colocalization-first approach and Kim's methodology. Nonetheless, there are discernible disparities worth noting. For instance, the obesity cluster is associated with elevated HOMA-IR in the Kim generated PGIs, but not in our approach. The ALP negative and hepatic glucose metabolism clusters seem to delineate separate pathways. In the case of ALP negative, it is evident in its link to higher ALT levels and reduced triglycerides. The liver lipid cluster is associated with elevated BMI in our cluster analysis, but this association is not mirrored in Kim's cluster.

The relationships observed between PGIs and clinical outcomes exhibit a combination of shared patterns and distinctions. Interestingly, none of the PGIs derived from Kim's methodology demonstrated a statistically significant association with chronic kidney disease. In contrast, the lipodystrophy cluster generated through our coloc approach displayed a notable association to an increased odds of chronic kidney disease.

In both studies the lipid and liver metabolism PGIs was associated with decreased cardiovascular disease and the lipodystrophic PGI associated with increased cardiovascular disease (as illustrated in Figure S3I). This reveals that while PGIs may yield comparable associations to metabolic outcomes, their relationships to clinical outcomes can indeed diverge. In conclusion, when it comes to predicting unique metabolic lab values, both approaches appear to perform comparably with only minor discrepancies in associations. However, it is challenging to perform a comparative analysis without starting with the exact same set of T2D variants. Further, it is difficult to establish a metric by which one can say one approach outperforms another. Instead, it can be stated that the coloc approach is a more stringent approach, which boasts the additional advantage: the capability to infer shared causal variants across GWAS. However, the approach of Kim et al is more computationally trackable with a larger number of GWAS and produces similar predictions.


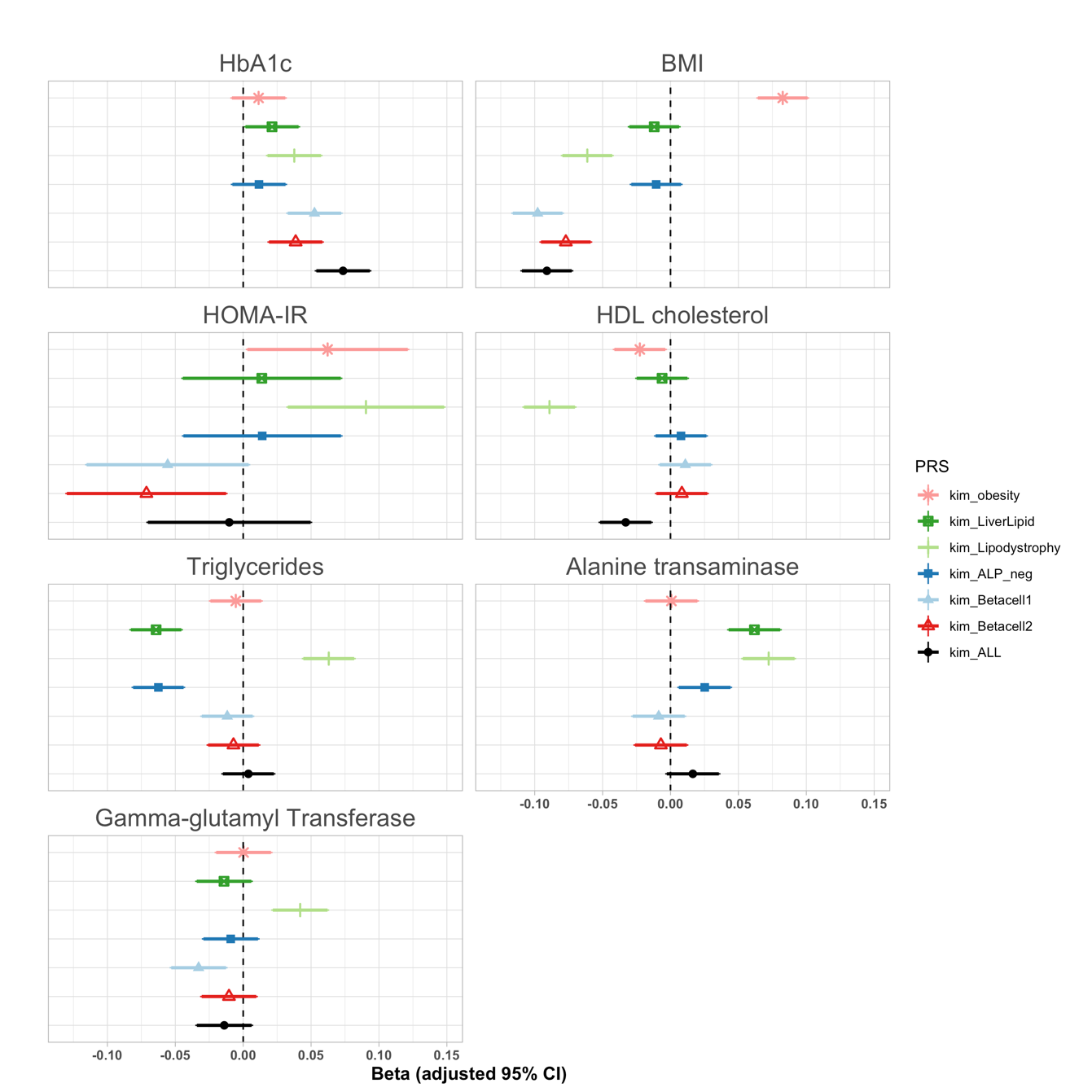

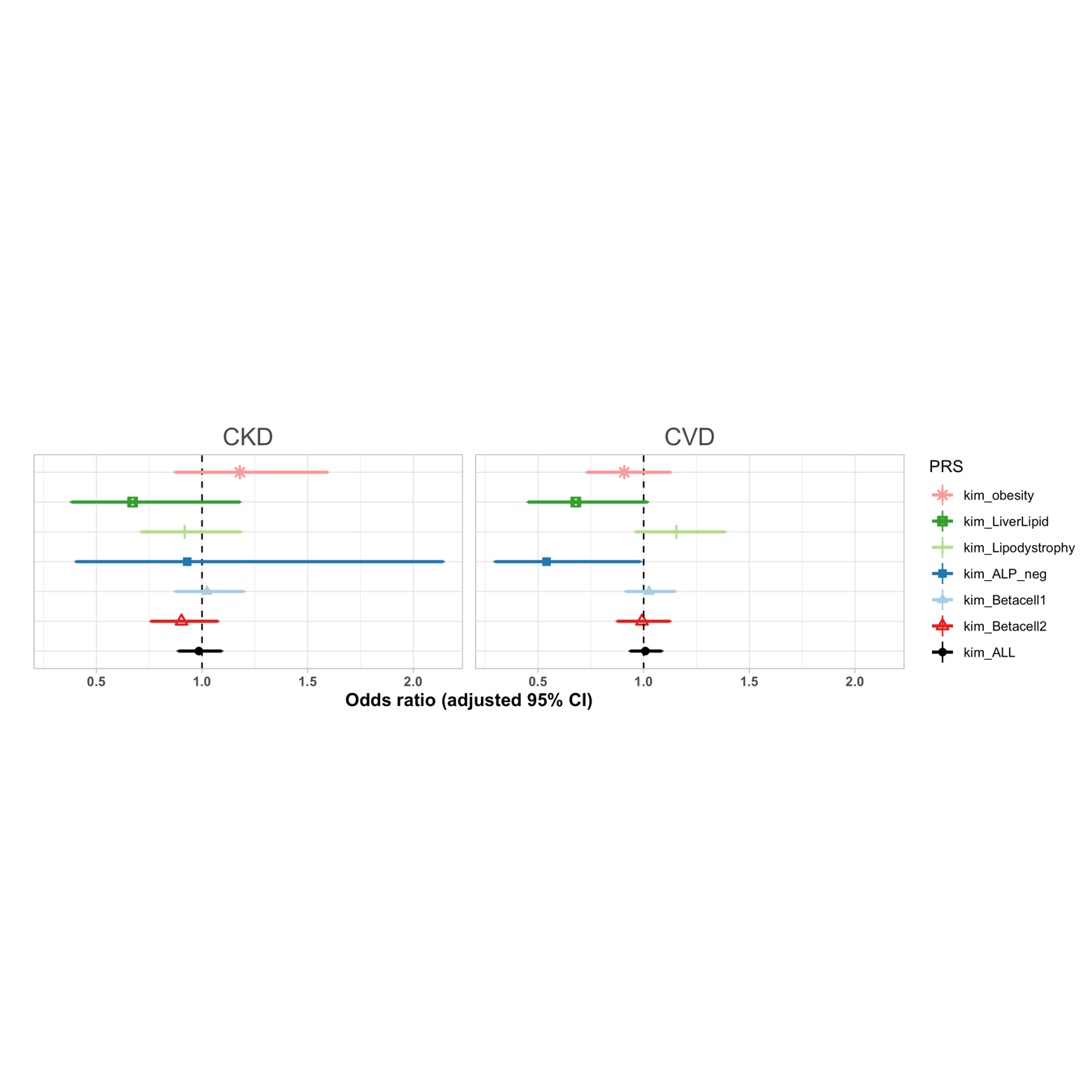


A

B

C

D

E

F

G

H

I

Figure S3 – Forest plots of associations of Kim et al pathway PGIs to metabolic measures in individuals with type 2 diabetes from three cohorts. A) HbA1C (n=18,517). B) BMI (n=21,281). C) HOMA-IR (Homeostatic Model Assessment for Insulin Resistance) (n=2,241). D) HDL cholesterol (n=19,370). E) Triglycerides (n=20,797). F) alanine transaminase (n=19,134). G) gamma-glutamyl Transferase (n=16,900). H) Chronic kidney disease (n=19,171). I) Cardiovascular disease (n=20,504). Linear regression was conducted for continuous outcomes (A,C,D,E,F,G) and logistic regression for binary (H,I) controlling for age, sex, BMI and cohort, besides BMI (B) which was controlled for sex and cohort. See Table S16 for the values underlying this figure.

Sensitivity analysis

To support robust colocalization results we opted to select traits with sufficiently large enough sample sizes (n >10,000). However, this biased the selection of GWAS more towards insulin resistance related traits and less insulin secretion related traits. To assess whether our results were biased in this regard we conducted sensitivity analyses. Colocalization was performed between T2D and a GWAS for oral glucose tolerance test (OGTT) with a sample size of 5,318. The genetic variants were then clustered and compared to the clusters generated without OGTT included. We observed significant evidence of colocalization with two genetic variants. The first 11:92708710 (H4.P = 1) also colocalized with HbA1C and fasting glucose. The second 15:62394264 (H4.P = 1) did not colocalize with any other of the metabolic traits tested. Four clusters were generated with a max correlation cut-off of 0.22. The beta-cell cluster (n=34), lipodystrophic insulin resistance (n=42), obesity (n=67) and hepatic glucose cluster (n=5). In general, the size of the clusters and the genetic variants within each cluster were similar to the results without OGTT. However, the lipid liver cluster was not present. This can be attributed to the lower maximum correlation cut-off, which made it challenging to classify these variants into distinct, separate clusters. Additionally, the beta-cell and obesity clusters gained some variants and the lipodystrophy lost some. The variants that switched clusters are likely the most pleiotropic ones, i.e., showing associations to multiple traits. These results highlight that the addition of a trait even with a small sample size can affect the clustering of variants. However, the resolution for distinguishing other clusters is hampered. Larger GWAS sample sizes are needed in order to provide a more balanced selection of traits that represent all major aspects of T2D heterogeneity.


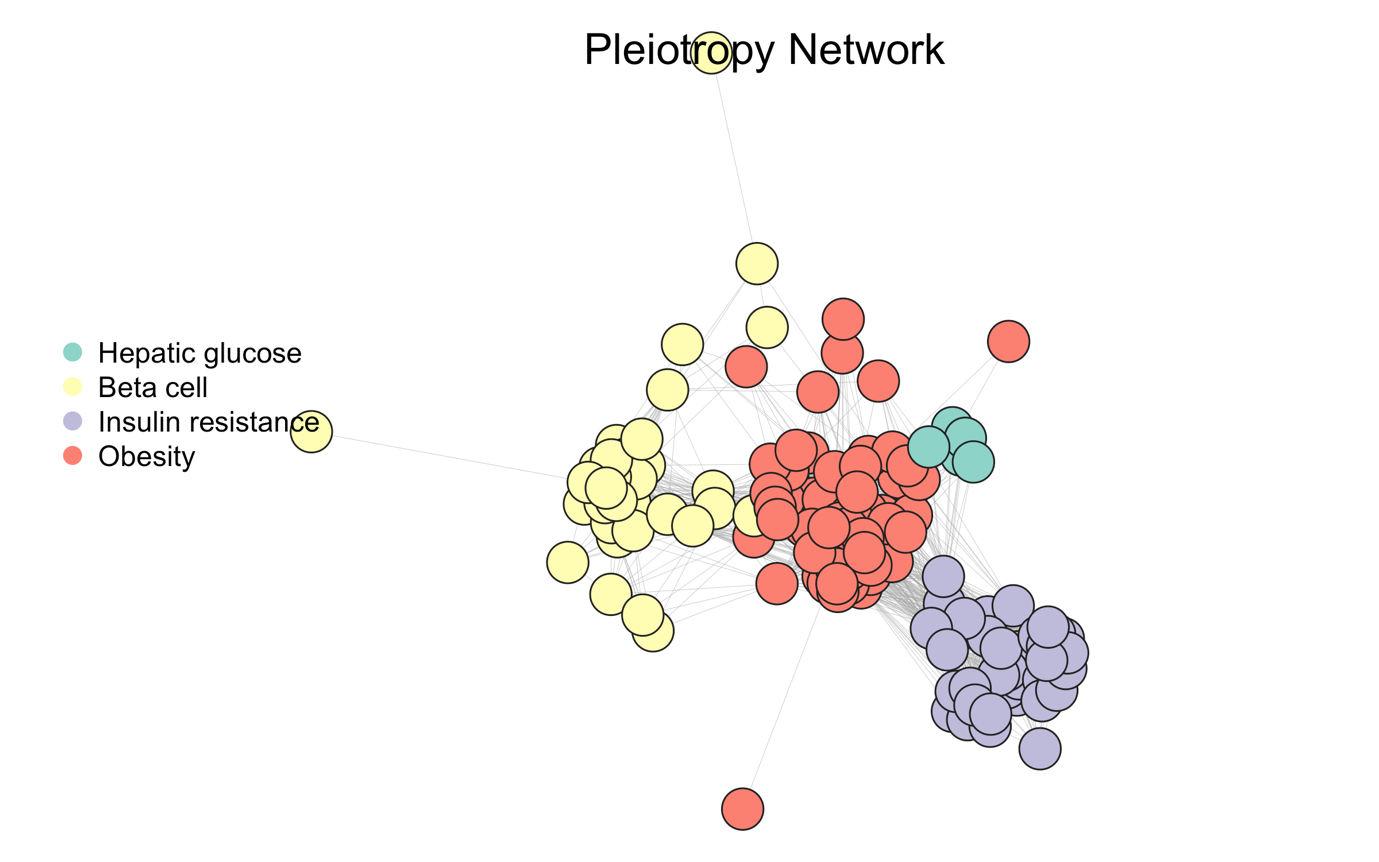


Figure S4 - Network analysis of T2D genetic variants. Variants were clustered according to their pleiotropic associations with related traits plotted into the network, with nodes representing SNPs and the edges the correlations between SNPs based on trait Z-scores. SNPs that shared similar associations with metabolic traits clustered together. Four clusters were identified relating to insulin resistance, beta-cell deficiency, obesity, hepatic glucose metabolism.
